# Instrumented gait analysis post‐anterior cruciate ligament reconstruction in pediatric patients: A non‐invasive method for quantifying the static and dynamic leg axis

**DOI:** 10.1002/jeo2.70320

**Published:** 2025-06-29

**Authors:** Leonie Kijewski, Eva Böker, Karoline Hofmann, Thomas Engel, Pierre Hepp, Maren Witt

**Affiliations:** ^1^ St. Elisabeth Hospital Leipzig Germany; ^2^ Department of Orthopedics, Trauma Surgery, and Plastic Surgery University Clinic Leipzig Leipzig Germany; ^3^ Department of Biomechanics Faculty of Sports Science at the University of Leipzig Leipzig Germany

**Keywords:** axis deviation, growth plate injury, instrumented gait analysis, pediatric ACL reconstruction, rehabilitation

## Abstract

**Purpose:**

This study explores the potential of Instrumented gait analysis (IGA) as a non‐invasive tool for monitoring rehabilitation in pediatric patients with anterior cruciate ligament (ACL) injuries. Current clinical assessments, such as physical exams and X‐rays, have limitations in evaluating dynamic knee alignment and loading. IGA may offer a more precise method to track rehabilitation progress and detect altered gait biomechanics following ACL reconstruction (ACLR). We hypothesize that IGA can provide insights into differences in frontal knee alignment and mechanical loading between static and dynamic conditions in pediatric patients following ACLR.

**Methods:**

IGA was conducted on 18 patients (mean age: 15 ± 2 years) at 3 and 12 months following ACLR, and seven conservatively treated patients (mean age: 12 ± 3) at 12 months post‐injury. Retroreflective markers were placed using the CAST lower body model. The gait was recorded in 3D using 12 infrared and two video cameras. Ground reaction forces were measured with force plates. Frontal knee alignment was assessed from kinematic data at four gait events: double‐limb stance, initial contact, loading response, and mid‐stance, identified by C‐Motion Visual3D Professional. Frontal knee joint moments were calculated based on inverse kinematic and dynamic in Newton‐meters per kilogram (Nm/kg) at the specified gait events. Repeated‐measures ANOVA was used to compare frontal knee alignment between static and dynamic conditions, while paired t‐tests assessed differences between injured and uninjured extremities.

**Results:**

In the surgical group, a significant increase in body height occurred between 3 and 12 months (*p* = 0.001) without notable changes in the static or dynamic frontal axis (*p* > 0.05). At 12 months post‐surgery, mean frontal knee alignment differed significantly between gait events (*p* < 0.001), showing a transition from valgus during stance to a nearly neutral axis during loading response. A significant difference was observed at initial contact, where the affected leg remained in greater valgus than the unaffected side (*p* = 0.026). Joint moments showed no significant differences between the healthy and affected sides (*p* > 0.05). No significant differences were found between the surgical and conservative groups.

**Conclusions:**

The findings of this study suggest that IGA can detect dynamic alignment deviations, supporting its potential as a complementary tool for monitoring knee function following pediatric ACL injuries. The absence of significant alignment changes over time indicates a low risk of surgery‐induced growth plate injury. While IGA may help identify alignment‐related risk factors for re‐rupture, its ability to accurately predict graft insufficiency remains unclear.

**Level of Evidence:**

Level II, prospective cohort study.

**Abbreviations**
ACL: anterior cruciate ligament
ACLR: anterior cruciate ligament reconstruction
IC: initial contact
IGA: instrumented gait analysis
LR: loading response
MSt: mid‐stance
OA: osteoarthritis

## INTRODUCTION

In recent years, the incidence of anterior cruciate ligament (ACL) ruptures among children and adolescents has increased notably [4, 11, 16, 27, 37, 39]. Current literature indicates that reconstruction is the preferred treatment to achieve improved outcomes and higher rates of return to sports [10, 12, 14, 26, 32, 39]. Although growth plate‐sparing techniques are available, the transepiphyseal method is predominantly endorsed, raising concerns about potential growth plate injuries [4, 10, 12, 14, 15, 26, 32, 37, 39]. Furthermore, pediatric patients exhibit high rates of re‐rupture and contralateral injury [9, 23, 28, 38, 39]. To effectively recognize and prevent these risks, an optimal rehabilitation process is necessary. In clinical practice, rehabilitation is typically monitored through physical examinations or frequent X‐ray imaging [39]. However, subtle axis deviations indicative of growth plate injury, as well as dynamic alignment changes, are not detectable through clinical examination or static imaging. Recognized risk factors for ACL graft rupture, such as dynamic valgus [3, 18, 24, 29], may therefore remain undiagnosed. Additionally, frequent X‐rays are not feasible for young patients due to radiation concerns, limiting clinicians' ability to assess the risk of re‐rupture or postoperative growth disturbances.

Instrumented gait analysis (IGA) offers a non‐invasive method for assessing both the static and dynamic leg axis [35]. By comparing alignment during specific gait events with alignment during stance, IGA can help identify axis deviations that predispose patients to ACL graft rupture. This renders IGA an ideal tool for the non‐invasive assessment of potential postoperative malalignment and for risk stratification [31]. Unlike static X‐ray imaging, IGA provides additional information on potential knee overloading due to pathological axis deviation [5, 20, 21, 34, 35]. This may further aid clinicians in assessing the risk of osteoarthritis (OA) development due to malalignment [13, 17].

Despite its advantages, the use of IGA in clinical practice, especially for pediatric patients, has been limited due to restricted accessibility and the need for specialized personnel. To our knowledge, it has not been applied to pediatric ACL rehabilitation. This study examines its feasibility for integration into clinical practice, aiming to complement existing assessments with biomechanical insights not provided by standard exams or imaging. Specifically, we investigate whether IGA can detect differences in knee alignment and mechanical loading during the rehabilitation period. We hypothesize that frontal knee alignment does not differ between static and dynamic conditions and is equal between the injured and uninjured leg 12 months postoperatively. Additionally, we hypothesize that ACL‐reconstructed patients will exhibit different gait mechanics and knee loading compared to conservatively treated patients 12 months post‐injury.

## MATERIALS AND METHODS

### Study design

In partnership with the Saxony Accident Insurance Fund, a prospective multicentric longitudinal study was conducted on pediatric patients who sustained ACL injuries during school‐related activities since 2019. Patients were treated at different facilities, with treatment decisions made independently by the treating physicians and not influenced by the study. Surgical or conservative treatment allocation was then accepted upon participation. Due to the multicentric design, no standardized rehabilitation protocol was applied for either treatment. Patients who underwent transepiphyseal ACL reconstruction (ACLR) participated in gait analyses at 3 and 12 months post‐surgery, while those treated conservatively were assessed at 12 months post‐injury. Follow‐up evaluations were conducted in the Gait Analysis Lab of the Department of Sports Biomechanics at the Faculty of Sports Science, University of Leipzig. Patients received individualized training plans during follow‐up visits, recommending home training 2–3 times per week. These plans were tailored to each patient's performance level, with load parameters (repetitions, sets, weight and holding time) provided as recommendations and adaptable as needed. Patients were advised to discontinue any exercise that caused pain and to seek expert advice if necessary.

### Ethics statement

The study was approved by the Ethics Committee of the Medical Faculty at Leipzig University (reference number 376/18‐ek, dated December 17, 2018). Informed consent was obtained from the legal guardians of all participants.

### Participants

Twenty‐five patients met inclusion criteria. Participants were categorized based on their treatment modality into either the surgical (*n* = 18) or the conservative group (*n* = 7).

Inclusion criteria:

■ Participants aged between 10 and 17 years
■ MRI‐confirmed ACL rupture
■ Completed follow‐up period of 12 months following surgery or injury

Exclusion criteria:

■ Concurrent ligament injuries or non‐isolated ACL tears
■ Prior surgeries on the affected knee
■ Injuries to the contralateral knee

### Gait analysis

**Figure 1:**
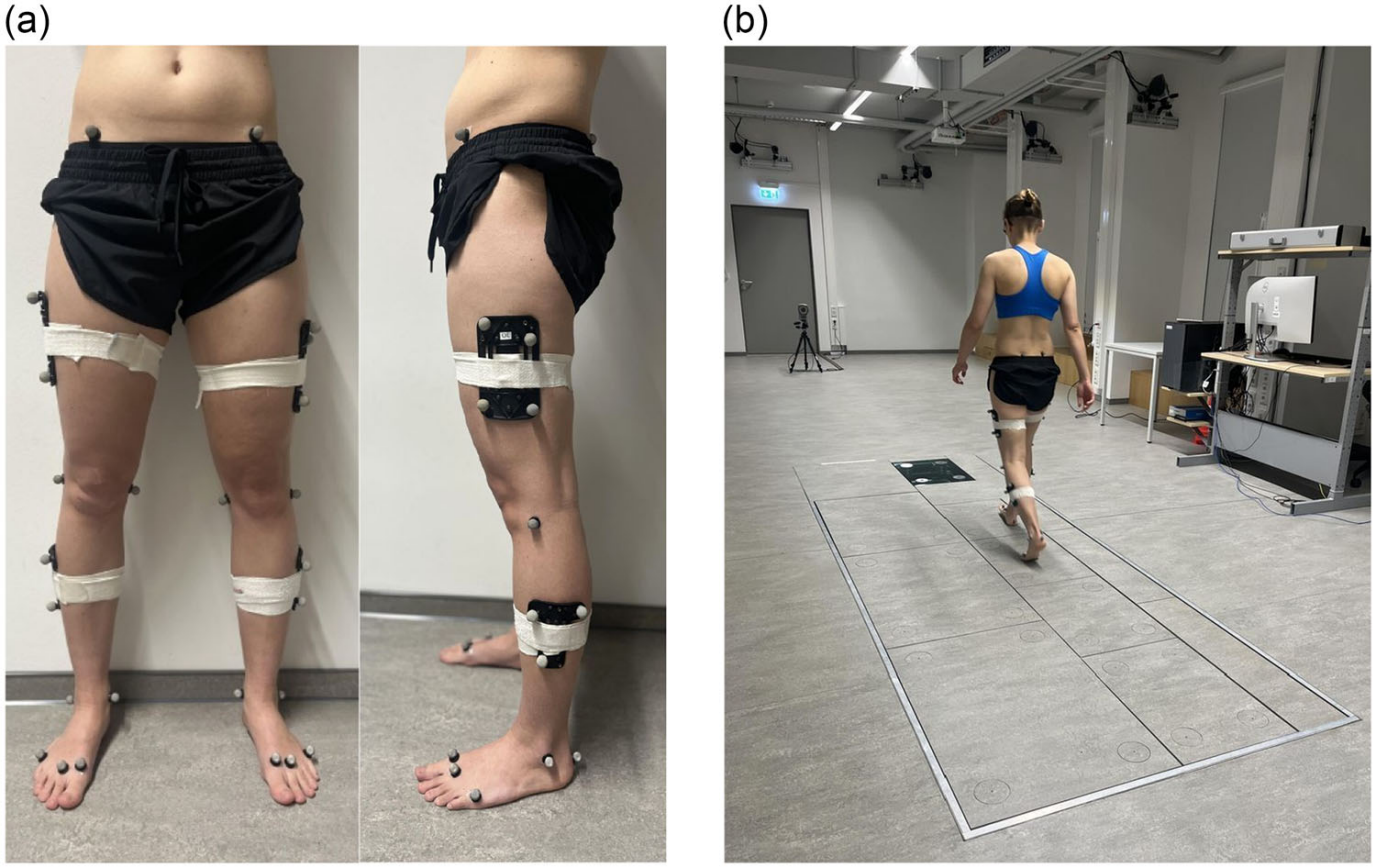
(a) Reflective marker placement using the CAST lower body model by Cappozzo et al. [8]. Twenty passive reflective markers are distributed across key anatomical landmarks, complemented by clusters of four markers on each thigh and three markers on each shin. Standardized markers of 14 mm diameter were utilized, with yellow markers designated for static measurements to ascertain the joint axis at the knees and ankles. Medial markers were subsequently removed for the dynamic gait trials. (b) Gait laboratory setup with Kistler Force Plates (Kistler Instrument AG, Winterthur, Switzerland). The configuration includes four small (Type 9281E) and three large plates (Type 9287C), accommodating different stride lengths to ensure accurate capture of each step.

**Figure 2:**
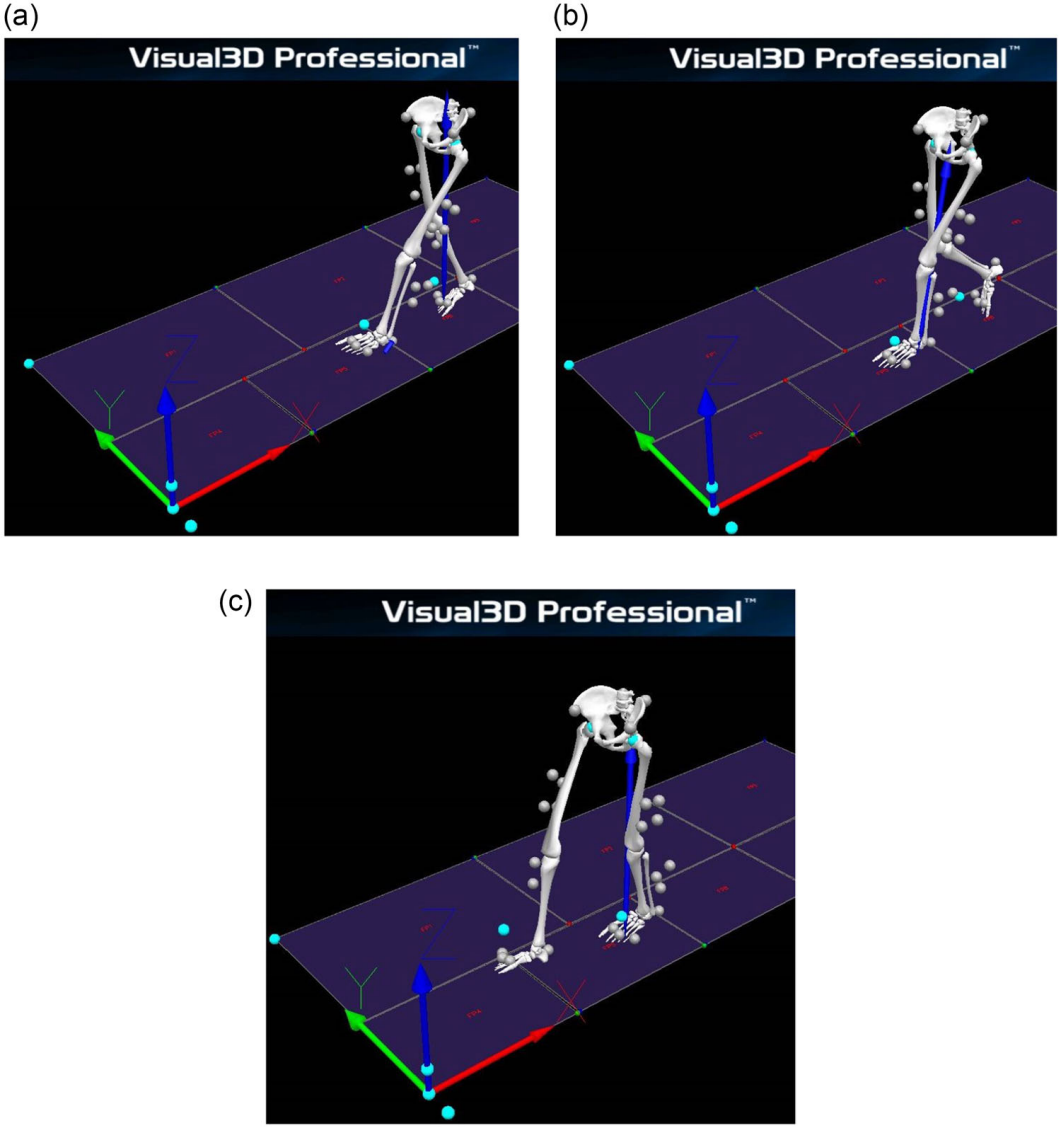
Identification of gait events in C‐Motion Visual3D Professional. (a) Initial contact (IC) marks the first ground contact of the measured extremity (left), entering the single support phase. (b) Loading response (LR) signifies the beginning of weight transfer as the opposite extremity (right) lifts off. (c) Mid‐stance (MSt) occurs when the body's weight is centralized over the stance limb, defined by the anterior‐posterior GRF crossing zero.

Demographic and anthropometric data were collected at each follow‐up visit (see Tables 1 and 2). To prepare for gait analysis, retroreflective markers were placed according to the CAST lower body model (see Figure 1a). The gait was recorded three‐dimensionally using 12 Qualisys Oqus 700+ infrared cameras and two video cameras. The data were then transmitted to Qualisys Track Manager software (Qualisys, Inc., Gothenburg, Sweden). A sampling frequency of 100 Hz was employed to track marker positioning. Ground reaction forces were measured with Kistler Force Plates (Kistler Instrument AG, Winterthur, Switzerland; see Figure 1b). Participants were required to complete a minimum of 10 valid trials walking a 10‐meter distance at a natural pace. Frontal knee alignment was assessed from kinematic data at four gait events: double‐limb stance (stance), initial contact (IC), loading response (LR) and mid‐stance (MSt) (see Figure 2). Frontal knee joint moments were calculated using inverse kinematics and dynamics in Newton‐meters per kilogram (Nm/kg) at the specified gait events.

**Table 1:** Patient characteristics surgical group (mean with standard deviation).

Variable	
*n*	18
Sex	Female (*n* = 8); male (*n* = 10)
Age, years at time of injury	15.2 ± 2.0
Height, m^a^	1.7 ± 11.3/1.7 ± 9.9
Weight, kg^a^	68.9 ± 16.4/71.4 ± 17.3
Body mass index, kg/m^2^ ^a^	23.8 ± 4.4/24.3 ± 4.8
Concomitant injuries	Medial meniscus (*n* = 3); lateral meniscus (*n* = 3); both menisci (*n* = 3); none (*n* = 9)

^a^ At 3‐month follow‐up/at 12‐mounth follow‐up.

**Table 2:** Patient characteristics conservative group (mean with standard deviation).

Variable	
*n*	7
Sex	Female (*n* = 4); male (*n* = 3)
Age, years at time of injury	12.4 ± 2.7
Height, m^a^	1.6 ± 12.2
Weight, kg^a^	54.7 ± 19.4
Body mass index, kg/m^2^ ^a^	20.5 ± 5.1
Concomitant injuries	Medial meniscus (*n* = 1); none (*n* = 6)

^a^ At 12‐mounth follow‐up.

A custom data pipeline was implemented in C‐Motion Visual3D Professional for automatic gait event detection. At least five valid gait cycles per participant were required. For each gait event within these cycles, 2–3 measurements were taken, resulting in approximately 10–15 measurements per event. The mean of these measurements was used for the final analysis.

### Statistical analysis

All statistical analyses were conducted using SPSS version 27.0.1.0, with statistical significance set at *p* < 0.05. Descriptive statistics were employed to summarize demographic data of the patient cohorts, with Shapiro–Wilk testing verifying normal distribution. Boxplots were examined to identify potential outliers. For the surgical group, paired *t*‐tests were performed to compare gait analysis results between 3 and 12 months postoperatively, as well as to compare the affected and unaffected joints at 12 months. Effect sizes were calculated using Cohen's *d*. Additionally, a repeated‐measures ANOVA was conducted to evaluate changes in knee alignment and joint moments during the gait cycle. A Greenhouse‐Geisser correction was applied due to a violation of sphericity, as identified by Mauchly's test. Bonferroni‐corrected post hoc tests were used for pairwise comparisons to analyze the main effects, and again Cohen's *d* was used to determine the effect size. Mann–Whitney *U* testing was applied to identify differences between the surgical and conservative groups due to unequal cohort sizes.

## RESULTS

### Postoperative differences in knee alignment and joint moments

Patients in the surgical group showed a significant increase in body height by an average of 1.4 cm (*p* = 0.001), with no significant changes in BMI (*p* = 0.16). Static and dynamic knee alignment remained unchanged in both extremities over time (*p* > 0.05).

In stance, knee alignment remained in valgus at 3 months (affected: −2.7 ± 4.7°, unaffected: −1.6 ± 5.0°) and at 12 months (affected: −2.8 ± 3.5°, unaffected: −1.8 ± 4.5°). At initial contact, knee alignment followed the same pattern, with valgus alignment observed on the affected side at 3 months (−0.8 ± 4.5°) and 12 months (−1.72 ± 3.23°), while the unaffected side showed values of −0.3 ± 5.0° and −0.5 ± 4.3°, respectively.

Similarly, no significant differences in static or dynamic joint moments were observed between time points for either leg (*p* > 0.05). At loading response, joint moments remained stable over time, with values of 0.2 ± 0.1 Nm/kg at 3 months and 0.3 ± 0.1 Nm/kg at 12 months on the affected side, and 0.2 ± 0.1 Nm/kg and 0.3 ± 0.1 Nm/kg on the unaffected side.

### Comparison of dynamic gait events and differences between injured and uninjured extremities

**Figure 3:**
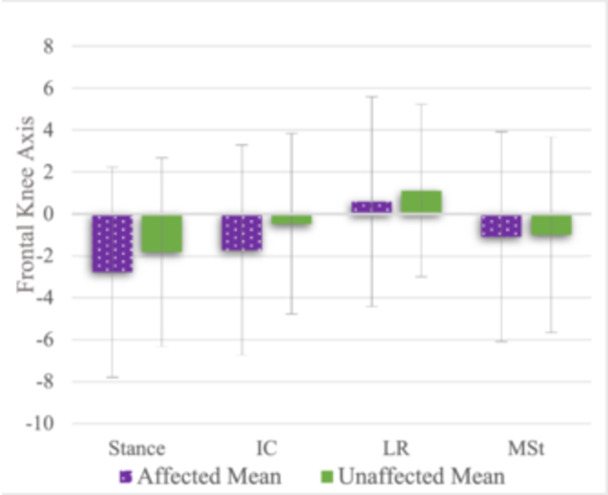
Comparison of knee alignment between the affected and unaffected sides across different gait events at 12 months in the surgical group. During the gait cycle, frontal alignment shifts from valgus during stance to a nearly neutral axis during loading response (LR), followed by another decline during mid‐stance (MSt). A significant difference was observed at initial contact (IC), where the affected leg remained in greater valgus compared to the unaffected side (*p* = 0.026). Positive y‐axis values represent varus, while negative values represent valgus alignment. Purple (dotted) represents affected means, while green (solid) represents unaffected means. Error bars represent standard deviations.

In the surgical group, significant changes in knee alignment were observed under dynamic conditions for both the affected [*F*(3, 51) = 12.94, *p* < 0.001, partial *η*² = 0.43] and unaffected legs [*F*(1.97, 33.46) = 11.06, *p* < 0.001, partial *η*² = 0.39]; see Figure 3. During the gait cycle, knee alignment shifted from valgus in stance [affected: −2.8°, unaffected: −1.8°] to varus in LR [affected: 0.6°, unaffected: 1.1°]; see Table 3. Bonferroni‐adjusted post‐hoc analysis revealed a shift toward varus alignment, with mean increases of 3.4° [95% confidence interval [CI] (1.35–5.39), *p* < 0.001] on the affected and 3.0° [95% CI (0.99–4.89), *p* = 0.002] on the unaffected leg. A significant difference between extremities was observed at initial contact, with the affected leg remaining in greater valgus than the unaffected side [affected: −1.7°, unaffected: −0.5°; *p* = 0.026, *d* = 0.57]; see Table 3.

**Table 3:** Mean frontal knee axis during stance and dynamic gait phases at the 12‐month follow‐up of the affected and unaffected leg in the surgical group.

Gait phase	Affected	Unaffected	Sig.	*d*
Stance, °	−2.8 ± 3.5	−1.8 ± 4.5	**0.113**	0.39
Initial contact (IC), °	−1.7 ± 3.2	−0.5 ± 4.3	**0.026**	0.57
Loading response (LR), °	0.6 ± 2.9	1.1 ± 4.1	0.410	0.19
Mid‐stance (MSt), °	−1.1 ± 3.5	−1.0 ± 4.7	0.881	0.04

*Note*: Bold values indicate a significant difference was observed at initial contact (IC), with the affected leg remaining in greater valgus compared to the unaffected side (*p* = 0.026).

**Figure 4:**
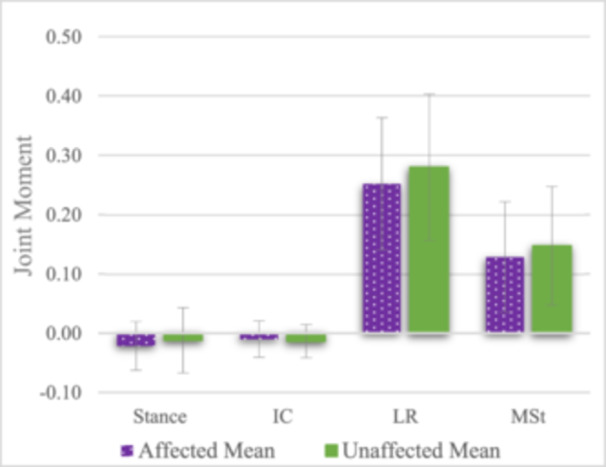
Comparison of joint moments between the affected and unaffected sides across different gait events at 12 months in the surgical group. A significant increase in joint moments, with the highest values observed during LR, was noted, with no significant difference between extremities. Purple (dotted) represents affected means, while green (solid) represents unaffected means. Error bars represent standard deviations. IC, initial contact; LR, loading response; MSt, mid‐stance.

In addition to alignment changes, knee loading significantly increased under dynamic conditions for both the affected [*F*(1.99, 33.96) = 81.68, *p* < 0.001, partial *η*² = 0.83] and unaffected legs [*F*(1.91, 32.45) = 63.93, *p* < 0.001, partial *η*² = 0.79]; see Figure 4. The highest joint moments were observed during LR, with mean increases of 0.3 Nm/kg [95% CI (0.20–0.34), *p* < 0.001] on the affected and 0.3 Nm/kg [95% CI (0.21–0.37), *p* < 0.001] on the unaffected leg compared to stance. Joint moments did not differ between extremities; see Table 4.

**Table 4:** Mean joint moments during stance and dynamic gait phases at the 12‐month follow‐up of the affected and unaffected leg in the surgical group.

Gait phase	Affected	Unaffected	Sig.	*d*
Stance, Nm/kg	−0.02 ± 0.04	−0.12 ± 0.05	0.41	0.19
Initial contact (IC), Nm/kg	−0.01 ± 0.03	−0.01 ± 0.03	0.64	0.11
Loading response (LR), Nm/kg	0.25 ± 0.11	0.28 ± 0.12	0.41	0.19
Mid‐stance (MSt), Nm/kg	0.13 ± 0.09	0.15 ± 0.10	0.48	0.17

### Comparison of surgical and conservative treatments

Mann–Whitney *U* tests revealed no significant differences between the surgical and conservative groups at the 12‐mounth Follow up. Specifically, there were no significant differences in static alignment of the affected or unaffected legs, nor in dynamic alignment across gait events (*p* > 0.05). Similarly, joint moments across gait events showed no significant differences between groups (*p* > 0.05).

## DISCUSSION

This study demonstrated that, despite a significant increase in body height, no significant changes in knee alignment or joint moments were observed over 12 months following ACL reconstruction in pediatric patients (*p* > 0.05). Knee alignment consistently followed a valgus‐to‐neutral pattern across the gait cycle, with greater valgus observed at initial contact on the affected side in the surgical group (*p* = 0.026). Additionally, no significant differences emerged between treatment groups (*p* > 0.05). These findings indicate that IGA can detect alignment differences beyond standard clinical assessments, showing potential as a complementary tool for evaluating knee function following pediatric ACL injury.

Concerns about potential growth plate injuries still affect treatment decisions in clinical practice, with frequent X‐ray imaging being the only option for clinicians to objectively assess the risk. While IGA has not yet been widely utilized to monitor postoperative growth disturbances in pediatric patients, the static leg axis, measured through IGA, has shown a strong correlation with radiological misalignment [35]. Rendering this method, a promising opportunity to assess alignment while minimizing radiation exposure, particularly in younger cohorts [33, 40]. In the present study, no significant surgery‐induced deviations in the frontal plane were observed, suggesting that concerns on growth plate injuries may be unwarranted [4, 15, 37, 39]. However, when applying this method, the impact of BMI on IGA accuracy must be considered, as a higher BMI may exaggerate valgus malalignment by obscuring skeletal landmarks with excess tissue [35]. The average BMI in this study remained below the threshold where correlation diminishes, reinforcing the reliability of the presented results.

Another key advantage of IGA over static imaging is its ability to measure dynamic alignment. For example, the presence of dynamic valgus deviation, a known risk factor for ACL re‐rupture in adults [3, 18, 24, 29, 30]. In this study, static and dynamic knee alignment were compared to determine whether alignment changes or remained consistent during the gait cycle. A shift from valgus during stance to a nearly neutral axis during LR was observed, followed by a decline into varus during MSt. This pattern aligns with normal gait mechanics, where varus loading increases internal stress on the medial compartment during the gait cycle [2, 19]. Notably, this trend was observed in both the injured and non‐injured extremities. However, at the 12‐month follow‐up, the affected extremity exhibited greater valgus alignment at IC. Since this valgus deviation did not progress throughout the gait cycle, it does not indicate dynamic valgus. Nevertheless, it could potentially increase the load on the ACL graft, as valgus alignment persisted during a critical phase of gait. Although the observed differences were minor and their clinical relevance remains uncertain, they may still highlight IGA's ability to detect subtle deviations that might be missed by standard clinical assessments [7]. The lack of significant changes in this pattern over time also suggests that the deviation is more likely a pre‐existing risk factor rather than a postoperative complication. Although these findings do not confirm a risk of re‐rupture, the use of IGA to assess dynamic alignment deficits shows great potential for identifying the underlying causes of high risk of graft rupture in pediatric patients [9, 23, 28, 38, 39]. Furthermore, young patients have exhibited different gait biomechanics following ACLR [27, 30, 36], underscoring the necessity to integrate IGA into long‐term rehabilitation programs to further investigate the biomechanics after pediatric ACLR.

In cases of dynamic axis deviation, IGA also assesses potential mechanical overloading, which is again not visible in static imaging [5, 16, 21, 35]. In this study, joint moments were highest during the LR, with the knee positioned near neutral alignment. As this occurred during a dynamic gait phase, static assessments would have failed to identify the knee position under the highest load. Dynamic analysis can thus help detect functional deficits and guide rehabilitation toward improving functional knee stabilization [7, 40]. Additionally, elevated frontal knee varus moments have emerged as predictive biomechanical markers for knee osteoarthritis (OA) in gait analysis studies [16, 21, 22, 34]. At 12‐months, joint moments were found to be equivalent between extremities. Given the minimal differences in alignment, this result is not unexpected. However, it is important to note that these findings are limited to the frontal plane. This study still recommends complementing static axis evaluation with dynamic analysis to provide a more comprehensive assessment of OA risk [20, 34], as prior research indicates that knee varus moments may not increase until 4–5 years after ACLR [16, 17, 22]. Thus, long‐term monitoring is essential to fully capture any biomechanical changes that may contribute to OA development [13].

Finally, the lack of significant differences between treatment groups in this study can likely be attributed to the short follow‐up period. Additionally, the small size of the conservative treatment group may have limited the ability to detect nuanced differences. While the results might suggest that both treatment methods yield comparable functional outcomes, existing research tends to favor surgical treatment, particularly for patients with high functional demands [10, 12, 14, 26, 32, 39]. The available medical records did not permit a qualitative analysis of the rationale behind the choice for surgical or conservative treatment in individual cases. Notably, the mean age in the conservative group was significantly lower compared to the operative group (12 ± 3 years vs. 15 ± 2 years). This tendency may again be attributed to concerns regarding growth plate injuries in very young patients. Additionally, the proportion of patients with associated meniscal injuries was markedly higher in the operative group (9 out of 18) compared to the conservative group (1 out of 6). Supporting meniscus healing is considered a clear indication for ACLR [25]. Due to the proven high rates of concomitant injuries in children [1, 6], an increase in surgeries can be anticipated. Thus, the growing need and preference for surgical treatment underscores the importance of advancing techniques for monitoring postoperative rehabilitation. Only future research will determine whether IGA can serve as a reliable tool to predict graft rupture risk and ensure optimal recovery. However, the development of markerless systems for gait analysis that integrate more easily into clinical practice presents a promising step toward achieving this goal.

## LIMITATIONS

This study acknowledges several limitations. The lack of comprehensive preoperative IGA data impedes the full assessment of potential surgery‐induced axis deviation. Additionally, only the frontal plane was evaluated. No gender analysis was conducted, limiting the generalizability of the findings across different gender identities. The small sample size, lack of healthy controls and imbalance between treatment groups further reduce the statistical power of the study. Variability in rehabilitation protocols among participants could have influenced outcomes, potentially introducing additional bias. Furthermore, only walking at a natural pace was analyzed, with no running data collected. Since return to sports often involves running and cutting movements, future studies should incorporate these activities to provide a more comprehensive assessment of knee function following ACL injury in children. Future studies with larger, more balanced cohorts, standardized rehabilitation protocols, and preoperative IGA assessments are needed to provide a clearer and more comprehensive understanding. Longer follow‐up periods will also be essential to detect meaningful changes over time.

## CONCLUSION

This is the first study to evaluate a young cohort using IGA post‐ACLR and injury over an extended period. The lack of significant alignment changes over time suggests a low risk of surgery‐induced growth plate injury. While IGA shows potential in identifying alignment‐related risk factors for re‐rupture, additional long‐term studies are needed to determine whether IGA can accurately predict graft rupture.

## AUTHOR CONTRIBUTIONS

All authors contributed to the conception and design of the study. Specific contributions are as follows: *Material preparation, data collection, and analysis*: Leonie Kijewski, Eva Böker, Karoline Hofmann, Maren Witt, and Pierre Hepp. *Formal analysis and investigation*: Leonie Kijewski, Eva Böker, and Karoline Hofmann. *Drafting the manuscript (first draft)*: Leonie Kijewski. *Writing, review, and editing*: Maren Witt, Pierre Hepp, Leonie Kijewski, Thomas Engel, Eva Böker, and Karoline Hofmann. *Funding acquisition*: Maren Witt. *Resources*: Maren Witt. *Supervision*: Maren Witt and Pierre Hepp. All authors have read and approved the final manuscript.

## CONFLICT OF INTEREST STATEMENT

The authors declare no conflicts of interest.

## ETHICS STATEMENT

This study was conducted in accordance with the principles outlined in the Declaration of Helsinki. Ethical approval was granted by the Ethics Committee of the Medical Faculty at Leipzig University (reference number 376/18‐ek, dated December 17, 2018). Written informed consent was obtained from the legal guardians of all participants for the collection and publication of the presented data.

## Supporting information

Supporting Information.

## Data Availability

The data set generated and analyzed during the current study is available at the following link: https://doi.org/10.5281/zenodo.14629216.
